# Supplementary material for: Temperate forests can deliver future wood demand and climate-change mitigation dependent on afforestation and circularity
Source: Nat Commun. 2025 Apr 25;16:3872. doi: 10.1038/s41467-025-58463-5 (PMC12032007; doi:10.1038/s41467-025-58463-5)
Supplement: Supplementary file 1 — Supplementary Information [file 41467_2025_58463_MOESM1_ESM.pdf]

Supplementary Information

Temperate forests can deliver future wood demand and climate-change mitigation dependent on afforestation and circularity

Eilidh J. Forster*[1], David Styles[1,2] & John R. Healey[1]
[1] School of Environmental and Natural Sciences, Bangor University, Bangor, Gwynedd, LL57 2UW, UK
[2] School of Biological & Chemical Sciences and Ryan Institute, University of Galway, Galway, H91 TK33, Ireland

Supplementary Methods 1

Exploratory framework integrating forest modelling and LCA to identify 'low regrets' climate-change mitigation solutions for forestry value chains

In a deeply uncertain system, it is not possible to identify an optimal solution. Instead, by taking an exploratory approach, such as 'robust decision-making', it is possible to identify system vulnerabilities and determine 'low regrets' solutions that are likely to achieve positive outcomes[1,2].

We have developed a framework, setting out an exploratory approach for identifying 'low regrets' climate solutions for forestry value chains by evaluating a range of plausible future scenarios, using integrated forest modelling and lifecycle assessment (LCA) (Supplementary Fig. 1). The approach could also be useful for land-use modelling more broadly.

This framework builds on detailed prospective dynamic LCA modelling of entire forest-wood value chains developed and explained in Forster et al.[3,4]. Crucially, the full downstream greenhouse gas (GHG) mitigation consequences of wood use and end-of-life management are considered - including carbon storage and material and energy substitution within a decarbonising future economy. Novel aspects of the framework developed in the present study (shown in pink on Supplementary Fig. 1) include calculating potential future wood supply deficit by comparing projected wood demand curves to wood supply from a range of modelled augmented and expanded national/regional forest management scenarios; and linking the supply deficit to marginal expansion of supply from other regions.

Framework description

Goal and Scope: The nature of the system being assessed is deeply uncertain, requiring an exploratory approach to identify 'low regrets' solutions, rather than attempting to seek an optimal solution. The study question(s) is defined on this basis. The functional unit that flows through the framework is a defined area of domestic forest (regional- or national- (i.e. landscape-) scale; in the present study this is 100,000 hectares of generic temperate forest). From this, a range of domestic forest management change and/or expansion scenarios are defined. Projected demand curves, and potential management changes to overseas forests (representing a marginal change in supply from other regions), are also defined. Key scenario parameters (outlined in Table 1) should be tailored to suit a specific study-context. An LCA scope diagram is also recommended for summarising the study boundary and improving methodological transparency (see Supplementary Fig. 2 for the LCA scope diagram for the present study).

Inventory analysis: The baseline and counterfactual domestic forest scenarios are modelled based on appropriate forest inventory data (for example the United States Forest Inventory and Analysis' (FIA) Nationwide Forest Inventory (NFI) data[5], UK National Forest Inventory[6], or Canadian National Forest Inventory (NFI)[7]), and relevant forest growth models (such as CBM-CFS3[8] or LANDIS[9]). From these modelled scenarios, forest carbon dynamics are calculated, including quantification of annual domestic wood supply. There is a soft link from the forest carbon modelling, linking domestic wood supply to a material flow (breakdown of wood products from the forest gate through the value chain via processing, use, recycling and end-of-life). Domestic wood supply is also used to calculate the wood supply deficit (or surplus) in comparison with the demand projection scenarios defined earlier during the 'Goal and Scope' phase. The supply deficit (or surplus) links to an overseas wood supply material flow (breakdown of wood products from the forest gate through the value chain via processing, use, recycling and end-of-life); and also soft links to overseas forest modelling of the scenarios defined earlier during the 'Goal and Scope' phase. In the present study, changes to

overseas forest carbon stocks were modelled simply based on a literature review to determine a series of plausible management changes for particular forest types (e.g. a specified reduction in harvest interval for boreal forests) that could supply the additional wood demanded by a shortfall in supply from temperate forests. This approach could be refined for future studies through development of a database of marginal consequences of increased wood demand (increased harvest pressure) across different forest types. Such a database could be populated through a meta-analysis of existing studies, or a combination of modelling and data-mining. Key wood value chain parameters (outlined in Supplementary Table 1) should be tailored to the specific study-context. In the present study, we considered future technology and decarbonisation trajectories as described in Forster et al. (2021) for a UK context, based on detailed net zero projections (UKCCC)[10]. Alternative data sources may include country-specific projections (e.g. other country net-zero analyses) and scenario outputs from Integrated Assessment Models such as GCAM[11].

Impact assessment: Lifecycle inventory components from both the domestic and overseas forest modelling and the domestic and overseas wood value chain inventories are converted to global warming potential (GWP) impact (measured in kg carbon dioxide equivalent, kg CO_2e). Although the present study focuses on global warming potential (GWP) impact of the counterfactual scenarios, other environmental impact categories can also be assessed.

Interpretation: By varying key parameters of interest in the scenarios, this provides insights into the sensitivity of those parameters to GWP impact. Hence, it can identify system vulnerabilities to successfully delivering climate-change mitigation; and can be used to identify ‘low regrets’ solutions i.e. actions that are likely to have a positive outcome. This in turn can be used to inform a coherent suite of supporting policy and action to drive positive change and impact.

Supplementary Methods 2

Modelling of natural disturbances – 'reduced productivity'

In this study we have taken the approach of testing multiple scenarios of variation in future forest productivity (yield) as an efficient way of proxying diverse drivers of uncertainty in yield and greenhouse gas (GHG) effects related to forest management practices.

We modelled four core management scenarios to test the impacts of modified rotation length ('reference-rotation', 'extended-rotation', 'shortened-rotation') and enhanced tree growth rate ('higher productivity') (Table 1, main paper). These are intended to capture uncertainties linked to intentional actions that may be taken by forest managers, e.g. altering harvest intensity, rotation length, and use of improved genotypes or new species (leading to higher growth rates and/or higher resistance to threats). In addition, although the effects of atmospheric CO_2 enrichment and climate warming leading to increased forest growth rates[12] are not directly modelled, their potential effects are indicated by the 'higher productivity' scenario.

Uncertain factors negatively affecting forest productivity that are not linked to intentional forest management decisions include natural disturbances (drought, fire, wind, pests and pathogens) and nutrient limitation, which lead to forest damage and reduced yield[12-16]. To evaluate the possible impact of forest damage caused by natural disturbances a further 'lower productivity' forest scenario was also modelled. However, to maintain clarity and avoid introducing too many results in the main paper, we have included the detailed results of this 'lower productivity' scenario in Supplementary Information. This is justified as the results do not alter the main findings of the paper.

In order to construct a 'lower productivity' scenario that is representative of a range of future natural disturbances we undertook an extensive literature review. The predominant natural causes of forest damage include wildfires, windthrow, and insect pest epidemics[17], varying in prevalence across regions. For example, in Canada, wildfires and insect epidemics have been a frequent subject of research[17,18], and in Europe windthrow damage had been a predominant research focus[17,19] until the major impacts of bark beetle epidemics interacting with droughts that have devastated spruce forests in central Europe during the past two decades[20-24]. Natural disturbance-related forest damage is expected to worsen in the future, partially as a result of global warming[20,25].

Linking insect damage with its effects on the growing stock of wood, and its flows (and value) is challenging[26]. Following insect-related tree damage and mortality, carbon uptake drops dramatically, and living biomass is transferred into dead organic matter pools that decay over many years. The impact on carbon dynamics can vary widely[25-29].

Combustion during wildfire releases carbon directly to the atmosphere[30,31], while subsequent tree mortality, soil carbon loss, elevated respiration, and lower carbon sequestration rates amplify the initial carbon losses[32,33].

Weather-related damage and events can also impact forest productivity. Drought can also increase prevalence of wild fire[34] and drought-stressed forest can be more vulnerable to insect pest damage[23]. Weather-related damage, including wind, also changes the regular flow of wood from forests to processing facilities by disrupting supply chains and causing downstream effects[35].

At a regional scale, multiple forest disturbance types can accumulate across a landscape and the resulting diverse combinations of forest age, species, stand structure, and recovery stages complicate carbon dynamics through space and time. In Europe, since the 1950s 46% of total wood

volume damage was caused by wind; forest fires were responsible for 24%; almost 20% was caused by bark beetles; other pests and pathogens accounted for 8%[24].

The impact of disturbances on forest condition and productivity is clearly complex and potentially significant. For example, in British Columbia (Canada) a period of high natural disturbance by insects and wildfire led to net primary production losses of 10% between 2000 and 2018[18]. In Europe, over the last 20 years, disturbance on average accounted for 16% of the mean annual harvest[24]. A literature review by Vacek et al. (2023)[36] found that frequent and destructive large-scale forest disturbances are one of the most significant consequences of climate change for forest productivity. They also found that species migration and changes in tree growth rate have substantial effects on forest carbon stocks – varying from -1% to +99% in Central and Northern Europe, and -12% to -49% in southern Europe.

Management response to moderate disturbance

Although an increasing occurrence of forest damage is expected as climate change progresses, there can also be counteractive forest management practices implemented to reduce impacts. For example, preventative measures to minimise height differences between stands can reduce the risk of wind damage[37]. Removing flammable understory material (e.g. by shrub clearing or prescribed burning) may reduce the risk of catastrophic wildfires[35,38]. Removing or avoiding high-risk species and establishing a diverse stand structure could improve resilience in the long term[39,40]. Infrastructure investments such as road networks and other forms of fire break can be included in management plans as a component of measures to control fire damage[41].

Proposed ‘reduced productivity’ scenario

In summary, it is clear that under future climate change uncertain and potentially severe disturbances to forest productivity, carbon stocks and wood supply (among other impacts) are likely, though management responses could moderate these impacts somewhat. Location is also an important factor in the prevalence of type, intensity, frequency and spatial extent of disturbance.

Taking these factors on-board, along with findings on the range of intensity and endurance of loss of yield that disturbances could cause, we have specified the following scenarios intended to bound the possible impacts of disturbances on productivity and GHG fluxes. A key consideration was the spatial scale of our modelled forest (100,000 ha) relative to the scale of different disturbance impacts reported in the reviewed literature.

Two ‘lower productivity’ scenarios were modelled, representing two degrees of disturbance intensity. The new scenarios are a modification of the 'reference-rotation' existing forest (Yield Class (YC)18, 50-yr rotation) + afforestation combination (see Table 1). Both involve a 33% reduction in YC from YC18 to YC12 after first harvest.

1) The area affected by the YC reduction ramps up over 15 years to 15% of the reference-rotation ‘existing forest’ area (starting in year 2023) and lasts for one forest rotation. This reduces annual wood production by 16% (relative to reference-rotation existing forest wood production across 100,000 ha), for a 15 year period between 2052-2077 (Table 1, and Supplementary Data 3 (tab ‘IRW_supply_demand (Aff50yrs)’, cells AS57:71)). The forest recovers to reference conditions after this.
2) The area affected by the YC reduction ramps up over 15 years to 30% of the reference-rotation ‘existing forest’ area (starting in year 2023) and lasts for one forest rotation. This reduces annual wood production by 32% (relative to reference-rotation existing forest wood

production across 100,000 ha), for a 15 year period between 2052-2077 (Table 1, and Supplementary Data 3 (tab 'IRW_supply_demand (Aff50yrs)', cells AT57:71)). The forest recovers to reference conditions after this.

The gradual ramp-up of the area impacted is representative of the progress of most insect and pathogen epidemics, and of the cumulative impact of successive fires or windstorms in a vulnerable forest landscape. The intensity of disturbance modelled (the YC reduction) reflects the middle of the range of the diverse major disturbance impacts reported in the literature we reviewed above[20,24,30,34]. The disturbance is assumed to impact the productivity of the affected area for the remainder of a forest rotation (i.e. until harvest at 50 years) followed by productivity recovery due to restocking with a more resilient forest (e.g. tree species selection and mixture). We acknowledge that, in practice, damaged areas may undergo salvage harvesting and may be replanted earlier, however equally impacts may continue beyond the current rotation. We therefore elected to implement the conservative median scenario of recovery of the affected area after harvesting/replanting at the planned rotation length. Based on the implementation of adaptive forest management[42,43] we have assumed that both the replanted forest and newly established forest would be planned and managed to be more resilient to future disturbances.

We recognise that the type of natural disturbance, the intensity and rate of impact, the duration of impact and management responses to these are complex, with highly variable outcomes for forest productivity and condition, including wood quality. Therefore, the options for scenario modelling available are vast. However, we believe the selected 'reduced productivity' scenarios represent an appropriate scale of impact indicative of the sort of yield loses that could be associated with increased natural disturbance events under increasing climate change at the large landscape scale (100,000 ha) tested in this study, as well as providing a different temporal disturbance pattern (i.e. impact followed by recovery) from the other varied-management scenarios modelled in the study; and therefore they provide further useful insights.

Results

When 30% of the existing (reference-rotation) forest area is impacted by disturbance (leading to a 32% reduction in annual harvest volume across the total 100,000 ha for a 15-yr period) this leads to a cumulative net loss of climate change mitigation of 12.7 Tg CO_2e over the modelled period of 100 years (equating to a 7% loss for the 'low demand - high expansion' scenario). When the area impacted by disturbance is 15% of the existing (reference-rotation) forest (leading to a 16% reduction in annual harvest volume across the total 100,000 ha for a 15-yr period), the cumulative net loss of mitigation is 6.3 TgCO_2e (equating to a 4% loss for the 'low demand - high expansion' scenario) (Supplementary Data 3).

As a comparison, 'Shortened-rotation' leads to a 23% loss of cumulative net climate mitigation compared to 'reference-rotation' (for the 'low demand - high expansion' scenario); and the 'lengthen-rotation' scenario results in a 2% improved climate change mitigation compared to 'reference-rotation' (for the 'low demand - high expansion' scenario) (Supplementary Data 3). If the disturbance had been applied to these modified rotation scenarios, the quantity of net mitigation loss would be similar to the 'reference-rotation' scenarios (since they are all YC18), with the forest recovery period for those scenarios reflecting the rotation period (45 or 55 years).

The 'higher-productivity' scenario leads to a 56% increase in cumulative net climate mitigation compared to the 'reference-rotation' (for the 'low demand – high expansion' scenario) (Supplementary Data 3). If the disturbance had been applied to the 'higher-productivity' scenario it

would be expected to have a higher relative loss of net mitigation than the altered-rotation scenarios due to higher harvest (domestic wood supply) losses but forest recovery following the disturbance would be quicker, reflecting the shorter 35-yr rotation period.

Given the spread of cumulative net climate-change mitigation impacts between the modified-rotation and higher-productivity scenarios (-23%, 2% and 56%, compared to ‘reference-rotation’ impact), the relatively small (-4% and -7%) disturbance impacts would not change the overall order of results since the same percentage area of reduced productivity would be applied across all scenarios. In fact, disturbance is likely to increase the spread of impact between the modified-rotation and higher-productivity scenarios.

Supplementary Fig. 1 - Exploratory framework integrating forest modelling and lifecycle assessment (LCA) to identify 'low regrets' climate-change mitigation solutions for forestry value chains

Refer to Supplementary Methods 1 for further details on application of the framework**.** LCIA means lifecycle inventory analysis. GWP is global warming potential – an environmental impact category measured in kg of carbon dioxide equivalent emissions.

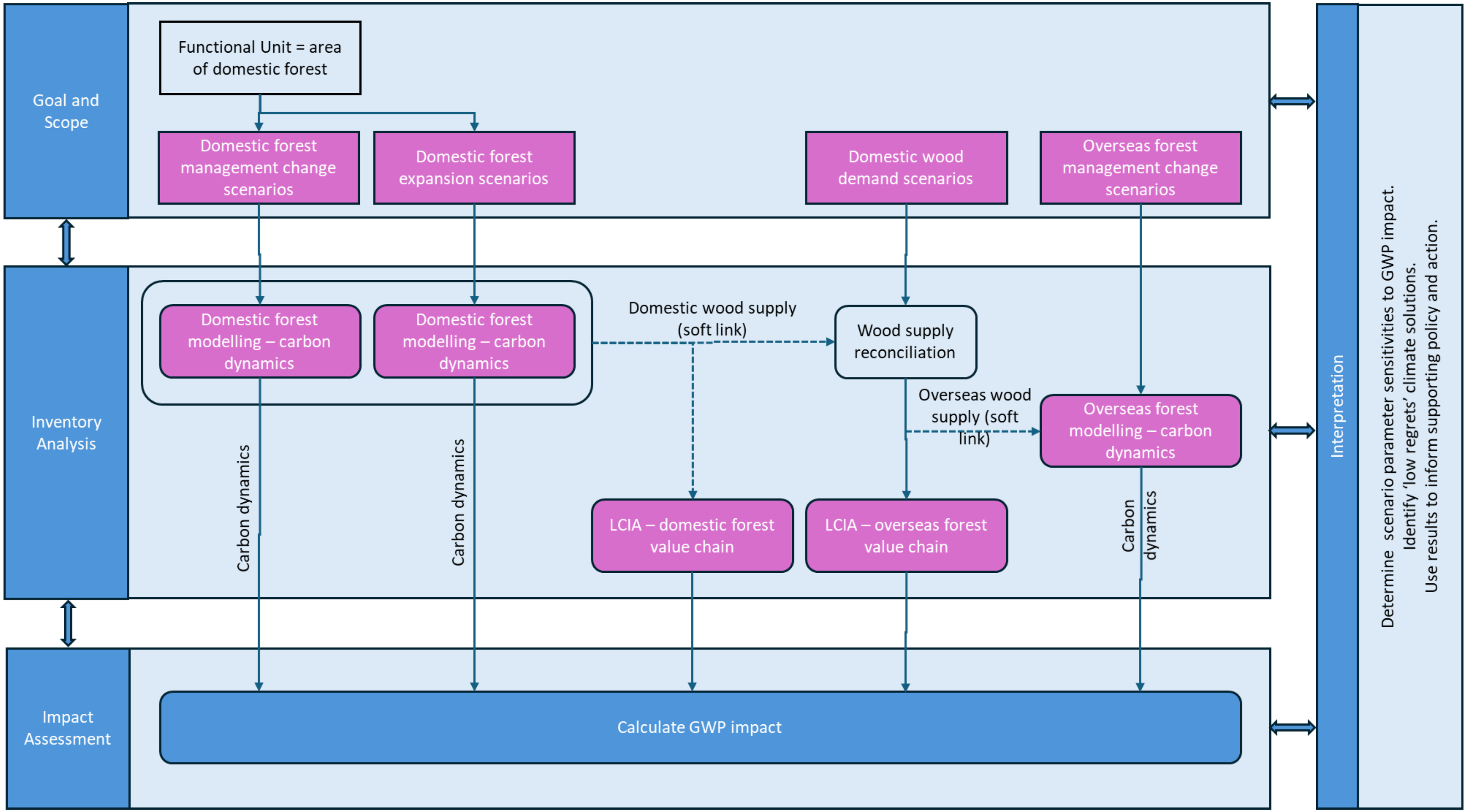

Goal and Scope
Functional Unit = area of domestic forest
Domestic forest management change scenarios
Domestic forest expansion scenarios
Domestic wood demand scenarios
Overseas forest management change scenarios
Inventory Analysis
Domestic forest modelling – carbon dynamics
Domestic forest modelling – carbon dynamics
Domestic wood supply (soft link)
Wood supply reconciliation
Overseas wood supply (soft link)
Overseas forest modelling – carbon dynamics
Carbon dynamics
Carbon dynamics
LCIA – domestic forest value chain
LCIA – overseas forest value chain
Carbon dynamics
Impact Assessment
Calculate GWP impact
Interpretation
Determine scenario parameter sensitivities to GWP impact.
Identify 'low regrets' climate solutions.
Use results to inform supporting policy and action.

Supplementary Fig. 2 – Lifecycle assessment system boundary (modified from Forster et al. (2021)[3]).

We calculate the GWP impact of system changes from year 0 'baseline' buisiness as usual (BAU) ‘existing forest’ value chain, due to changes in ‘existing forest’ management and area (‘new forest’) and in ‘overseas forest’ management to meet projected wood demand increases. ‘Existing forest’ refers to changes to forest management in existing temperate forests in the focal country. ‘New forest’ refers to afforestation in the focal country. ‘Overseas forest’ refers to changes to harvest intensity from tropical and boreal forests to make up marginal demand shortfalls from temperate forests. See Figure 1 and Table 1 in the main article for further details of modelled scenarios. Harvested wood product (HWP) substitution (‘avoided products’) is indicated in orange/yellow. ‘Avoided products’ are determined as the marginal product type directly substitutable with the HWP in question. Increased use of sawn wood in construction following forestry expansion is assumed to replace masonry walls. Specifically, a timber-frame structural wall design typical of a family home, replaces an equivalent specification concrete block wall (see Supplementary Table 1). Bioenergy replaces marginal sources of heat and electricity generation that change on a decadal basis according to a ‘Core’ decarbonisation pathway as elaborated in Forster et al (2021)[3], which aligns with the UK Committee on Climate Change Core decarbonisation pathyway[10]. Notably, natural gas remains the marginal heat and power source likely to be substituted by additional dispatchable bioenergy from wood harvested from forests. We assume that deployment of carbon capture and storage technology (CCS) occurs in parallel for fossil fuels and bioenergy. This means that uncertainty over the scale of future CCS deployment is reduced because long-term carbon sequestration via BECCS is inversely related to energy substitution credits as CCS reduces the carbon intensity of substituted fossil energy.

Conservatively, we attribute no substitution credits to HWP where wood is the dominant feedstock, including fence posts, paper, mulch, other short-lived products and boards. Consequently, whilst HWP carbon storage is modelled for these wood uses, we are likely to underestimate substitution credits, especially for the hierarchical scenarios.

Cascading wood product flows are indicated by the outputs of ‘Wood Recycling’, and include particle board manufacturing, paper and paperboard production and bioenergy.

LCA BOUNDARY – changes from BAU management of existing forest in year 2023

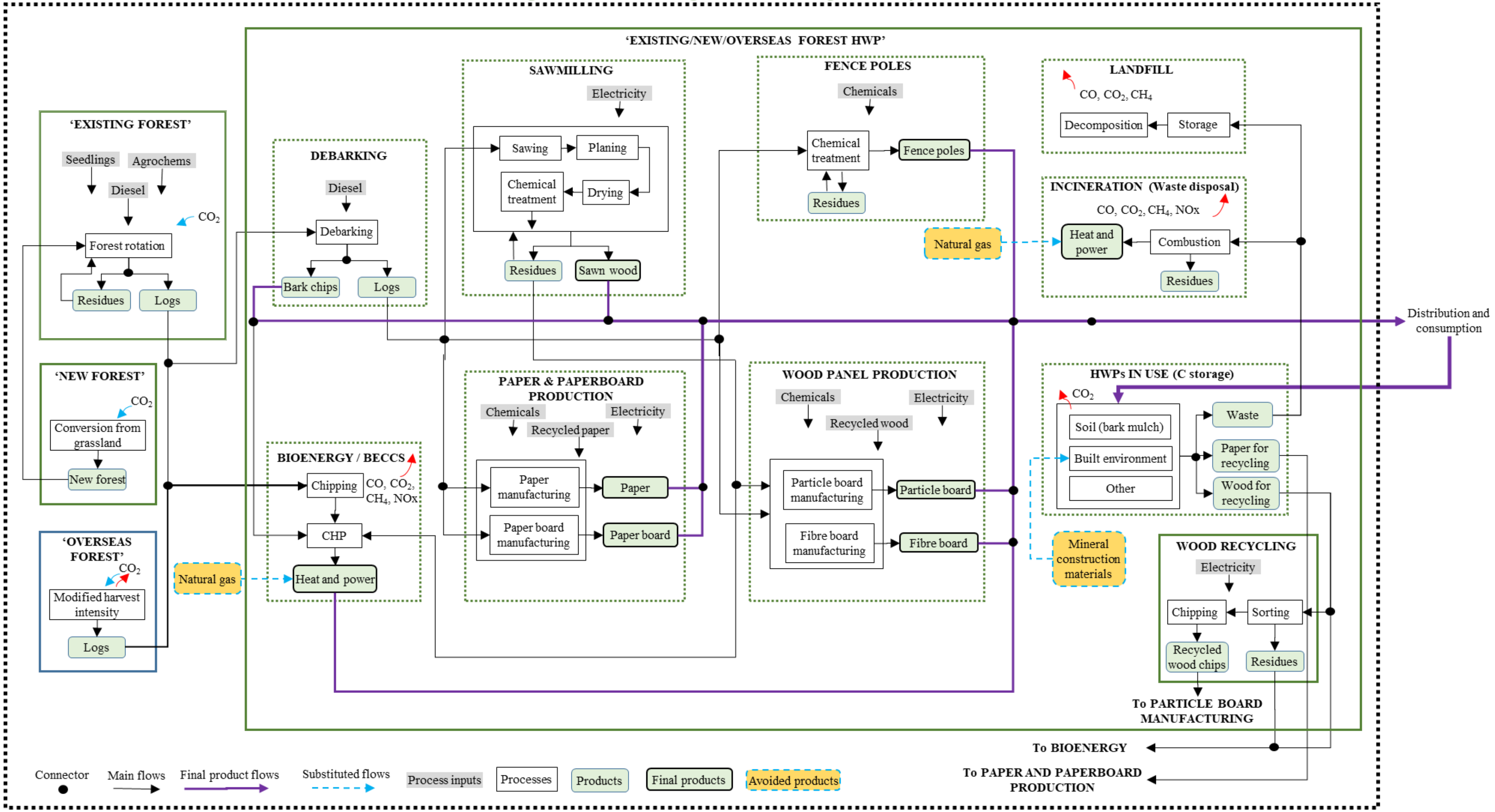

Supplementary Table 1 - Key forestry value chain parameters. These parameters should be tailored to represent specific study context.

Key value chain component	Key variables/parameters/assumptions (non-exhaustive)	Note	This study
Wood demand	Baseline demand		Continuation of year 0 demand (i.e. 0% annual increase)
	Counterfactual demand curves(s)	Range of plausible demand curves	Range of two % increases, based on published studies and data
Domestic forest (wood supply)	Area	Increasing disaggregation of species and forest types increases representation of landscapes but increases modelling complexity	Landscape scale – generic
	Afforestation rate		Range of afforestation rates – tested
	Age-class distribution (forest inventory data)		Even distribution of tree ages, i.e. age-classes
	Species/growth rate		Range of growth rates – tested
	Harvest intensity/rotation length		Range of harvest rotation timescales – tested
	Harvest practice (e.g. thinning, selective, clearcut)		No harvest, and thinning + clear cut
	Product breakout (at forest gate)	Can be broken down to different forest products at the forest gate (pulpwood, sawlogs etc.)	Five forest product categories
	Other	Consider the effects of climate warming, CO_2 fertilisation, albedo	Not assessed
Overseas forest (wood supply)	Area	Increasing disaggregation of species and forest types increases representation of landscapes but increases modelling complexity. Overseas forest may be impacted if the focal country is an exporter or importer of wood.	Landscape scale
	Afforestation rate		Afforestation rates varied according to demand – tested
	Age-class distribution (forest inventory data)		
	Species/growth rate		Range of forest types – tested
	Harvest intensity/rotation length		Range of harvest practices – tested
	Harvest practice (e.g. thinning, selective, clearcut)		Range of harvest practices – tested
	Product breakout (at forest gate)	Could be broken down to different forest products at the forest gate (pulpwood, sawlogs etc)	Assume same as domestic forest

Key value chain component	Key variables/parameters/assumptions (non-exhaustive)	Note	This study
	Supply deficit	Calculated as difference between domestic wood supply and wood demand	
Wood processing	Types of technologies / manufacturing processes	Reflects current and plausible future scenarios, considering technology development and market factors	Representative of temperate developed countries
	Wood product material flow (to consumer/use)	Different product breakouts (could be different for different harvest practices/forest types/geographical contexts)	Representative of temperate developed countries
	Fuel mix (of energy supply)	Representative of geographical context	Representative of UK
Transport	Transport/technology types	Reflects current and plausible future scenarios, given technology development and market factors	Representative of temperate developed countries
	Fuel type		Representative of temperate developed countries
	Transport distances		Representative of UK
Product substitution	(Fossil/mix) energy substitution	Representative of geographical context (and/or those published in peer reviewed scientific, or reputable grey literature), including current and plausible future scenarios, given technology development and market factors	Representative of UK
	Non-wood product substitution		Representative of UK
Product 'retiral' rate		IPCC assumptions (or other reputable peer reviewed scientific literature) or other realistic rate if testing variable. Could test the impact of increasing product life.	IPCC assumptions and peer reviewed scientific literature
Recycling rates	% of 'waste' wood collection and recycling	Reflect current rates or other future rates if testing this parameter	Representative of UK
	Cascading use(s) of 'waste' wood	Reflect current uses or other future uses if testing this parameter	Representative of UK
Decarbonisation	Rate of decarbonisation of electricity	Dynamic - apply dynamically to wood value chain and substituted product/energy value chains, representative of geological context	Recommended by UK Committee on Climate Change (UK CCC)
	Rate of decarbonisation of heat supply		UK Industry roadmaps
	Rate of decarbonisation of transport		Recommended by UK CCC
	Rate of other relevant industrial decarbonisation (e.g. cement production)		UK Industry roadmaps

Supplementary Table 2 - Life Cycle Inventory for 'business as usual' (BAU) wood use

Inventory of key inputs and outputs for processes considered along the life cycle of forestry value chains derived from thinned forest systems over 100 years. Emissions factors (EF) and their sources are indicated. GWP is global warming potential (measured in kg CO_2e).

Process stage	Input/output/process	Activity data source	Units	Thinned		EFs	EF source
				In	Out	GWP	
Planting (1&2)	Tree seedlings	GH[45]	Item(s)	774,012,298		0	Ecoinvent[44]
	15 tonne 360 Excavator	GH[45]	hrs	464,407		65	Ecoinvent[44]
	Pesticides (acetamiprip)	Industry recommended	kg	25,759			Ecoinvent[44]
Forest management	Harvester (diesel use)	GH[45]	hrs	1,207.459		56	Ecoinvent[44]
	Forwarder (diesel use)	GH[45]	hrs	1,207,459		46	Ecoinvent[44]
	Harvested wood	CBM-CFS3[46]	m^3		13,751,783		IPCC
Transport (forest to processor)	>32 t truck, EURO6	GH[45]	t.km	1,909,601,142			Ecoinvent[44]
Debarking	Harvested wood	CBM[46], GH[45]	m^3	11,634,008			
	Diesel	Ecoinvent[44]	MJ	10.250,052			
	Lubricating oil	Ecoinvent[44]	kg	9,095			
	Bark chips	GH[45], FR CFs[48]	kg		865,458,517	20	Ecoinvent[44]
	Debarked wood	GH[45], FR CFs[48]	m^3		10,387,507		
Sawing	Diesel (internal transport)	Ecoinvent[44]	MJ	105,021,592			
	Electricity	Ecoinvent[44]	kWh	70,229,933			
	Lubricating oil	Ecoinvent[44]	kg	382,034			
	Debarked wood	GH[45], FR CFs[48]	m^3	7,305,635			
	Sawnwood	JJ&S[47]	m^3		4,018,099	25	Ecoinvent[44]
	Sawmill residues	JJ&S[47]	kg		1,141,285,057		
Drying (of sawn timber)	Electricity	Ecoinvent[44]	kWh	67,102,253			
	Sawnwood	JJ&S[47]	m^3	4,018,099			
	Sawnwood - dried (u=20%)	Assume no loss in volume during drying	m^3		4,018,099	29	Ecoinvent[44]
Planing	Electricity	Ecoinvent[44]	kWh	34,840,025			
	Sawnwood (carcassing) dried (u=20%)	JJ&S[47]	m^3	4,018,099			
	Sawnwood (carcassing) planed	Vol loss accounted for in 'sawing'	m^3		4,018,099	35	Ecoinvent[44]
	Sawmill residues	JJ&S[47]	kg		1,141,285,057		
Chemical treatment	Electricity	Ecoinvent[44]	kWh	708,223			
	Wood preservative	Ecoinvent[44]	kg	991,511,510			
	Sawnwood (fencing) dried (u=20%)	JJ&S[47]	kg	688,296,981			
	Debarked wood (fence poles)	GH[45],FR CFs[48]	kg	303,214,529			
	Preserved wood	No vol. change	kg		991,511,510	0	Ecoinvent[44]
Particle board production	Electricity	Ecoinvent[44]	kWh	345,191,890			
	Heat	Ecoinvent[44]	MJ	4,050,362,146			
	Resin	Ecoinvent[44]	kg	169,276,792			
	Debarked wood (chip)	GH[45]	kg	596,750,357			
	Sawmill residues	JJ&S[47]	kg	700,595,575			
	Recycled wood	FC report	kg	1,108,766			
	Particle board	FR CFs[48]	m^3		3,415,000	262	Ecoinvent[44]
Fibre board production	Electricity	Ecoinvent[44]	kWh	685,822,000			
	Heat	Ecoinvent[44]	MJ	4,757,462,000			
	Debarked wood (chip)	GH[45] FR CFs[48]	kg	596,750,357			
	Sawmill residues	JJ&S[47]	kg	647,847,106			
	Fibre board	JJ&S[47], GH[45], FR CFs[48]	m^3		1,370,000	98	Ecoinvent[44]
Woodchip production (for biomass energy)	Electricity	Ecoinvent[44]	kWh	71,358,404			
	Lubricating oil	Ecoinvent[44]	kg	5,642			
	Harvested wood - 'fuel'	GH[45]	kg	765,829,625			
	Recycled wood - 'biomass'	FC	kg	2,000,000,000			
	Wood chips	GH[45]	kg, dry		2,765,829,625	0	Ecoinvent[44]

Process stage	Input/output/process	Activity data source	Units	Thinned		EFs	EF source
				In	Out	GWP	
Biomass energy	Electricity	Ecoinvent[44]	kWh		20,284,594,466		Ecoinvent[44]
	Wood chips	GH[45]	Kg, dry	2,765,829,625			Conversion biogenic C to CO_2 eq
	Heat	Ecoinvent[44]	MJ		52,550,762,866	0	Ecoinvent[44]
Avoided fossil fuels	Electricity generation (natural gas, high pressure)	Ecoinvent[44]	m^3	3,146,931,268			Ecoinvent[44]
Avoided construction materials	140 mm concrete block and mortar wall replaced by timber frame wall	BRE[49]	m^2	18,779		37	Ecoinvent[44]

Supplementary References

1 Workman, M. Darch, G., Dooley, K., Lomax, G., Maltby, J., Pollitt, H. Climate policy decision making in contexts of deep uncertainty - from optimisation to robustness. Environmental *Science & Policy* **120**, 127-137 (2021).

2 Workman, M., Darch, G., Denisart, B., Roberts, D., Wilkes, M., Brown, S., Kruitwagen, L. A robust decision-making approach in climate policy design for possible net zero futures. *Environmental Science & Policy* **162**, 103886 (2024).

3 Forster, E.J., Healey, J.R., Dymond, C. & Styles, D. Commercial afforestation can deliver effective climate change mitigation under multiple decarbonisation pathways. *Nature Communications* **12**, 3831 (2021).

4 Forster, E.J., Healey, J.R., Newman, G. & Styles, D. Circular wood use can accelerate global decarbonisation but requires cross-sectoral coordination. *Nat Commun* **14**, 6766 (2023).

5 US Forest Service (US FS). Nationwide Forest Inventory (NFI). Available from Forest Inventory and Analysis (FIA) DataMart. https://research.fs.usda.gov/products/dataandtools/tools/fia-datamart (Accessed 06/12/24)

6 Forest Research. National Forest Inventory. Available from https://www.forestresearch.gov.uk/tools-and-resources/national-forest-inventory/ (Accessed 06/12/24)

7 National Forest Inventory (NFI). Canada's National Forest Inventory. Available from https://nfi.nfis.org/en/ (Accessed 06/12/24)

8 Kurz, W. A. et al. CBM-CFS3: A model of carbon-dynamics in forestry and land-use change implementing IPCC standards. *Ecological Modelling* **220**, 480–504 (2009).

9 Wang, W., He, H. & Fraser, J., Thompson, F., Shifley, S., Spetich, M.. LANDIS PRO: A landscape model that predicts forest composition and structure changes at regional scales. *Ecography* **37** 225-229 (2014).

10 UK CCC. *Net Zero: The UK's contribution to stopping global warming*. https://www.theccc.org.uk/wp-content/uploads/2019/05/Net-Zero-The-UKs-contribution-to-stopping-global-warming.pdf (2019).

11 (GCAM) Documentation for Global Change Analysis Model (GCAM). Project maintained by Joint Global Change Research Institute. https://jgcri.github.io/gcam-doc/v4.2/toc.html (Accessed 06/12/24)

12 Petersson, H., Ellison, D., Mensah, A.A., Berndes, G., Egnell, G., Lundblad, M., Lundmark, T., Lundström, A., Stendahl, J. & Wikberg, P-E. On the role of forests and the forest sector for climate change mitigation in Sweden. *Global Change Biology Bioenergy* **14**, 793-813 (2022).

13 Metsaranta, J.M., Shaw, C.H., Kurz, W.A., Boisvenue, C. & Morken, S. Uncertainty of inventory-based estimates of the carbon dynamics of Canada's managed forest (1990–2014). *Canadian Journal of Forest Research* **47**, 1082-1094 (2017).

14 Pau, M., Gauthier, S., Boulanger, Y., Ouzennou, H., Girardin, MP. & Bergeron, Y. Response of forest productivity to changes in growth and fire regime due to climate change. *Canadian Journal of Forest Research* **53**, 663-676 (2023).

15 Brecka, A.F.J., Boulanger, Y., Searle, E.B., Taylor, A.R., Price, D.T., Zhu, Y.Y., Shahi, C. & Chen, H.Y.H.
Sustainability of Canada's forestry sector may be compromised by impending climate change. *Forest Ecology and Management* **474**, 118352 (2020).

16 Dale, V.H. et al.Climate change and forest disturbances: Climate change can affect forests by altering the frequency, intensity, duration, and timing of fire, drought, introduced species, insect and pathogen outbreaks, hurricanes, windstorms, ice storms, or landslides. *Bioscience* **51**, 723-734 (2001).

17 Roos, A. Forest damage and forest supply chains: a literature review and reflections *International Journal of Forest Engineering* **34**, 330-339 (2023).

18 Giles-Hansen, K. & Wei, X.H. Cumulative disturbance converts regional forests into a substantial carbon source. *Environmental Research Letters* **17**, 044049 (2022).

19 Hanewinkel, M., Hummel, S. & Albrecht, A. Assessing natural hazards in forestry for risk management: a review. *Eur. J. Forest Res*. **130**, 329–351 (2011).

20 Seidl, R. et al. Forest disturbances under climate change. *Nature Climate Change* **7**, 395–402 (2017).

21 Marini, L., Økland, B., Jönsson, A.M., Bentz, B., Carroll, A., Forster, B., Grégoire, J.-C., Hurling, R., Nageleisen, L.M., Netherer, S., Ravn, H.P., Weed, A. & Schroeder, M. Climate drivers of bark beetle outbreak dynamics in Norway spruce forests. *Ecography* **40**, 1426-1435 (2017).

22 Hlásny, T., König, L., Krokene, P. et al. Bark beetle outbreaks in Europe: State of knowledge and ways forward for management. *Curr. Forestry Rep.* **7**, 138–165 (2021).

23 Jactel, H., Petit, J., Desprez-Loustau, M-L., Delzon, S., Piou, D., Battisti, A. & Koricheva, J. Drought effects on damage by forest insects and pathogens: a meta-analysis. *Global Change Biology* **18**, 267-276 (2012).

24 Patacca, M., Lindner, M., Lucas-Borja, M.E., Cordonnier, T., Fidej, G., Gardiner, B., Hauf, Y., Jasinevičius, G., Labonne, S., Linkevičius, E., Mahnken, M., Milanovic, S., Nabuurs, G.-J., Nagel, T.A., Nikinmaa, L., Panyatov, M., Bercak, R., Seidl, R., Sever, M.Z.O., Socha, J., Thom, D., Vuletic, D., Zudin, S. & Schelhaas, M.-J. Significant increase in natural disturbance impacts on European forests since 1950. *Global Change Biology* **29**, 1359-1376 (2023).

25 IPCC. Climate Change 2022: Mitigation of Climate Change. Working Group II Contribution to the Intergovernmental Panel on Climate Change Sixth Assessment Report (AR6). IPCC, p. 2913 (2022).

26 Mathey, A.H. & Nelson, H. Assessing forest management strategies under a mountain pine beetle attack in Alberta: exploring the impacts. *Can J For Res*. **40**, 597-610 (2010).

27 Brown, M. G., Black, T. A., Nesic, Z., Foord, V. N., Spittlehouse, D.L., Fredeen, A.L., Grant, N.J., Burton, P.J, & Trofymow, J.A. Impact of mountain pine beetle on the net ecosystem production of lodgepole pine stands in British Columbia. *Agric. Forest Meteorol.* **150**, 254–64 (2010).

28 Brown, M., Black, T.A., Nesic, Z., Fredeen, A.L., Foord, V.N., Spittlehouse, D.L., Bowler, R., Burton, P.J., Trofymow, J.A., Grant, N.J. & Lessard, D. The carbon balance of two lodgepole pine stands

recovering from mountain pine beetle attack in British Columbia. *Agric. Forest Meteorol.* **153**, 82-93 (2012).

29 Mathys, A., Black, T.A., Nesic, Z., Nishio, G., Brown, M., Spittlehouse, D.L., Fredeen, A.L., Bowler, R., Jassal, R.S., Grant, N.J., Burton, P.J., Trofymow, J.A. & Meyer, G. Carbon balance of a partially harvested mixed conifer forest following mountain pine beetle attack and its comparison to a clear-cut. *Biogeosciences* **10**, 5451–63 (2013).

30 Meigs, G.W., Donato, D.C., Campbell, J.L., Martin, J.G. & Law, B.E. Forest fire impacts on carbon uptake, storage, and emission: the role of burn severity in the Eastern Cascades, Oregon. *Ecosystems* **12**, 1246–67 (2009).

31 Amiro, B.D., Todd, J.B., Wotton, B.M., Logan, K.A., Flannigan, M.D., Stocks, B.J., Mason, J.A., Martell, D.L. & Hirsch, K.G. Direct carbon emissions from Canadian forest fires, 1959–1999 *Can. J. For. Res.* **31**, 512–25 (2001).

32 Dore, S., Montes-Helu, M., Hart, S.C., Hungate, B.A., Koch, G.W., Moon, J.B., Finkral, A.J. & Kolb, T.E. Recovery of ponderosa pine ecosystem carbon and water fluxes from thinning and stand-replacing fire. *Glob. Change Biol.* **18**, 3171–85 (2012).

33 Bond-Lamberty, B., Wang, C. & Gower, S.T. Net primary production and net ecosystem production of a boreal black spruce wildfire chronosequence. *Glob. Change Biol*. **10**, 473–87 (2004).

34 Grünig, M., Seidl, R. & Senf, C. Increasing aridity causes larger and more severe forest fires across Europe. *Glob. Change Biol.* **29**, 1648–1659 (2023).

35 Craighead, C.W., Blackhurst, J., Rungtusanatham, M.J. & Handfield, R.B. The severity of supply chain disruptions: design characteristics and mitigation capabilities. *Decision Sci*. **38**, 131–156 (2007).

36 Vacek, Z., Vacek, S. & Cukor, J. European forests under global climate change: Review of tree growth processes, crises and management strategies. *Journal of Environmental Management* **332**, 117353 (2023).

37 Zubizarreta-Gerendiain, A., Pukkala, T. & Peltola, H. Effects of wind damage on the optimal management of boreal forests under current and changing climatic conditions. *Can. J. Forest Res*. **47**, 246–256 (2017).

38 Ryan, K.C., Knapp, E.E. & Varner, J.M. Prescribed fire in North American forests and woodlands: history, current practice, and challenges. *Frontiers in Ecology and the Environment* **11**, e15-e24 (2013).

39 Garcia-Gonzalo, J., Pukkala, T. & Borges, J.G. Integrating fire risk in stand management scheduling. An application to Maritime pine stands in Portugal. *Ann. Oper. Res.* **219**, 379–395 (2014).

40 Dymond, C.C., Tedder, S., Spittlehouse, D.L., Raymer, B., Hopkins, K., McCallion, K. & Sandland, J. Diversifying managed forests to increase resilience. *Can. J. Forest Res*. **44**, 1196–1205 (2014).

41 Brack, C.L. & McLarin, M. Strategic forest planning and operational decisions under uncertainty. *Australian Forestry* **80**, 1–9 (2017).

42 Lawrence, A. Adapting through practice: Silviculture, innovation and forest governance for the age of extreme uncertainty. *For. Policy Econ.* **79**, 50–60 (2017).

43 Nagel, L.M., Palik, B.J., Battaglia, M.A., D’Amato, A.W., Guldin, J.M., Swanston, C.W., Janowiak, 901 M.K., Powers, M.P., Joyce, L.A., Millar, C.I., Peterson, D.L., Ganio, L.M., Kirschbaum, C. & Roske, M.R. Adaptive Silviculture for Climate Change: A National Experiment in Manager-Scientist Partnerships to Apply an Adaptation Framework. *J. For.* **115**, 167–178 (2017).

44 Wernet, G. et al. The ecoinvent database version 3 (part I): overview and methodology. *Int. J. Life Cycle Assess*. **21**, 1218–1230 (2016).

45 Gresham House. Forest production data. (2018).

46 Kull, S. J., Northern Forestry Centre (Canada) & Canada. Natural Resources Canada. Operational-scale carbon budget model of the Canadian forest sector (CBM-CFS3) : version 1.2, user’s guide. (2016).

47 James Jones & Sons. Sawmill production data provided from personal correspondence. (2019).

48 Matthews, R. W., Jenkins, T. A. R., Mackie, E. D. & Dick, E. C. Forest Yield: A handbook on forest growth and yield tables for British forestry. (2016).

49 BRE. IMPACT database v5 (accessed via etool LCA software). (2018).
