# Supplementary material for: Temperate forests can deliver future wood demand and climate-change mitigation dependent on afforestation and circularity
Source: Nat Commun. 2025 Apr 25;16:3872. doi: 10.1038/s41467-025-58463-5 (PMC12032007; doi:10.1038/s41467-025-58463-5)
Supplement: Supplementary file 2 — Description of Additional Supplementary Files [file 41467_2025_58463_MOESM2_ESM.pdf]

Description of Additional Supplementary Files

File Name: Supplementary Data 1
Description: LCA Calculation file

File Name: Supplementary Data 2
Description: Harvested wood products GWP impact calculation file

File Name: Supplementary Data 3
Description: LCA results synthesis file
